# Sorghum crops classification and segmentation using shifted window transformer neural network and localization based on (YOLO)v9-path aggregation network

**DOI:** 10.3389/fpls.2025.1586865

**Published:** 2025-09-23

**Authors:** Javaria Amin, Rida Zahra, Alena Maryum, Amber Sarwar, Amad Zafar, Seong-Han Kim

**Affiliations:** ^1^ Department of Computer Science, Rawalpindi Women University, Rawalpindi, Pakistan; ^2^ Department of Computer Science, University of Wah, Wah Cantt, Pakistan; ^3^ Department of Computer Science, National University of Technology (NUTECH), Islamabad, Pakistan; ^4^ Department of Artificial Intelligence and Robotics, Sejong University, Seoul, Republic of Korea

**Keywords:** transformer neural network, sorghum, YOLO-v9, hyperparameters, crops, classification, localization, and segmentations

## Abstract

**Introduction:**

The world’s population has been increasing continuously, and this requires prompt action to ensure food security. One of the top five cereals produced worldwide, sorghum, is a staple of the diets of many developing nations. For this reason,getting accurate information is crucial to raising cereal productivity. The quantity of crop heads arranged in various branching configurations can be used as an indicator to estimate the yields of sorghum. For various crops, computerized methods have been demonstrated to be beneficial in automatically collecting this information. However, the application of sorghum crops faces challenges due to variations in the color and shape of sorghum.

**Methods:**

Therefore,a method is proposed based on the three models for the classification, localization, and segmentation of sorghum. The shifted window transformer (SWT)network is proposed to have seven layers of path embedding, two Swin Transformers, global average pooling, patch merging, and dense connections. The proposed SWT is trained on the following selected hyperparameters: patch size(2,2), two window size,1e-3 learning rate,128 batch size,40 epochs, 0.0001 weight decay, 0.03dropout, eight heads, 64 embedding dimension, and 256 MLP. To localize the sorghum region, the YOLOv9−c model is trained from scratch on the selected hyperparameters for 100 epochs. Due to light, illumination, and noise, the sorghum images are more complex. A transformer-based SegNetmodel is designed, in which features are extracted using a pre-trained SegFormer-B0 model fine-tuned for ADE-512-512. The proposed model is trained from scratch for 10 epochs using the Adam optimizer with a learning rate of 5e-5 and CrossEntropyLoss hyperparameters, which are finalized after extensive experimentation to achieve more accurate segmentation of the sorghum; this is a significant contribution of this work.

**Results and Discussion:**

The achieved outcomes are superior to those in other published works.

## 1 Introduction

Global crop production is facing various climate challenges. These challenges include high temperatures and extreme weather conditions. These extreme situations lead to damage to crops (Liaqat et al., 2024). The crops are subject to different biotic and abiotic impacts, which can affect production and pose a threat to the agricultural economy. Various kinds of crops are used in abundance for different uses, which include food, feed, and fuel. Sorghum is a model crop for tropical grasses. It is best known for addressing these conditions, and it is essential to meet all nutritional needs (Baloch et al., 2023). Despite that, most Western countries use sorghum primarily as animal feed. The powerful ability of sorghum crops to resist harsh climate challenges can make them a key ingredient in a healthy diet (Khoddami et al., 2023). Sorghum is rich in fiber, protein, and essential minerals, making it an excellent ingredient in a variety of foods. The lipid content of sorghum is low, but it contains a high level of acid. Along with these, it also contains vitamins B and E and essential minerals such as phosphorus, magnesium, iron, and zinc. It can also lower the risk of chronic disease due to its unique phytochemical composition (Tanwar et al., 2023). Despite the many benefits of sorghum crops, the slow breeding methods and complexity of the environment reduce the urgency of production. The research focus is to enhance the production of sorghum as a climate-smart crop in global crop production. Phenotyping is essential for improving crops; however, a research gap that hinders progress exists (Hivare et al., 2024).

As crop diseases can be a global threat to food production worldwide, early detection of crop diseases helps decrease poor production and increase the quality and quantity (Ngugi et al., 2024). Advancements in the fields of image processing and machine learning, along with their applications in agriculture, are making exceptional progress. The deep-learning-based solution can facilitate the early detection of crop diseases and enhance accuracy through timely predictions (Bouacida et al., 2024). Although there is work done for the classification of sorghum crops, there is very little work present for the identification of sorghum disease detection. The core contribution steps of this research are as follows:

▪ Three models are proposed for classification, localization, and segmentation.
▪ The SWIN model is proposed based on the selected layers and optimal hyperparameters to classify the different types of sorghum plant images.
▪ To localize the actual sorghum region, ResNet50 was applied as the backbone for feature extraction and then passed to YOLOv9-c, PANet, and the detection head. The proposed YOLOv9-PANet model is trained from scratch to optimize hyperparameters for accurate bounding box prediction and classification.
▪ The transformer-based SegNet model is designed and trained from scratch on selected hyperparameters to segment the sorghum region more accurately. The proposed SegNet model comprises the SegFormer encoder and SegFormer decoder head. The encoder contains patch embedding, convolutional, and normalization layers, among others, while the decoder head comprises a segformer-MLP, linear projection, dropout, and classifier.

The article is organized as follows: Section II discusses the related work, Section III explains the proposed method’s steps, and Section IV presents the results and discussion. The conclusion is provided in Section V.

## 2 Related work

In the research, a small unmanned system UAS is used to gather high-resolution images, which prove to be efficient in the large crop field (Latif et al., 2021). A model is presented for the identification of diseases such as tar spot, anthracnose, and rust on leaves using a deep-learning-based ResNet architecture. A benchmark dataset is curated for the experiment. The methodology used image masking to focus on disease-related features. The proposed model demonstrated the accuracy reported in (Varur et al., 2024). Another significant disease that affects sorghum crops is charcoal rot sorghum (CRS). EfficientNet B3 and a fully convolutional network achieved a high accuracy rate of 86.97% in detecting CRS. For the segmentation, the FCN showed an accuracy level of 97.6%. The experiment showed increased validation scores with the increase in the size of image patches (Gonzalez et al., 2024). A convolutional neural network based on AlexNet is used for sorghum detection. The model achieves an accuracy rate of 97% (Senthil Kumar et al., 2023). A rapid and nondestructive model, which is based on hyperspectral imaging HIS technology, is used to detect pesticide residues in sorghum. The experimental data consisted of one group of sorghum treated with pesticide and three groups without pesticide treatment. The model obtained an accuracy level of 97.8% (Hu et al., 2024). In the research, a cloud-based deep learning algorithm is used. The phenotype traits are extracted with the segmentation. Manually measured phenotype traits are used to validate the extracted phenotypic traits. The model named PointNet++ demonstrated the best performance, achieving an accuracy rate of 91.5% (Patel et al., 2023). The smartphone-captured images are used to detect the sorghum panicle and grain number estimation. The images captured through the smartphone were all manually labeled and augmented. The models based on Detectron2 and YOLOv8 were trained. Both showed an accuracy level of 75% and 89%, respectively (Santiago et al., 2024). A novel approach, Split-Attention Networks, is employed for disease detection using aerial images. A pixel-based approach is used to classify each pixel as health or disease-prone. The proposed model achieved an F1-score of 89.04% (Divya et al., 2024).

The YOLOv8 detector is utilized for detecting the fall armyworm pest at early stages to prevent damage and enhance crop safety (Mhala et al., 2024). The DINO transformer (Swin backbone with ResNet-101), detection transformer (DETR), YOLOv8, EfficientNet B4, and WeedSwin Transformer models are designed for weed detection (Islam et al., 2025). Detection models such as Faster R-CNN with FPN, YOLOv5, and YOLOv7 are used to classify and detect the different types of coccinellids found in sorghum (Wang et al., 2023). The model is designed with the combination of YOLOv8s, the Gold module, and LSKA attention to enhance the detection of sorghum spikes (Qiu et al., 2024). UNet model with ResNet-34 as a feature extractor is used for segmentation and obtained a testing F1-score of 89% (Genze et al., 2022). The VGG-16 model is integrated with the attention module and channel-wise spatial convolution module into U-Net, providing an F1-score of 0.78 for crop classification. This model performs better than traditional U-net and Deeplabv3+ models (Yan et al., 2025).

## 3 Proposed methodology

**Figure 1:**
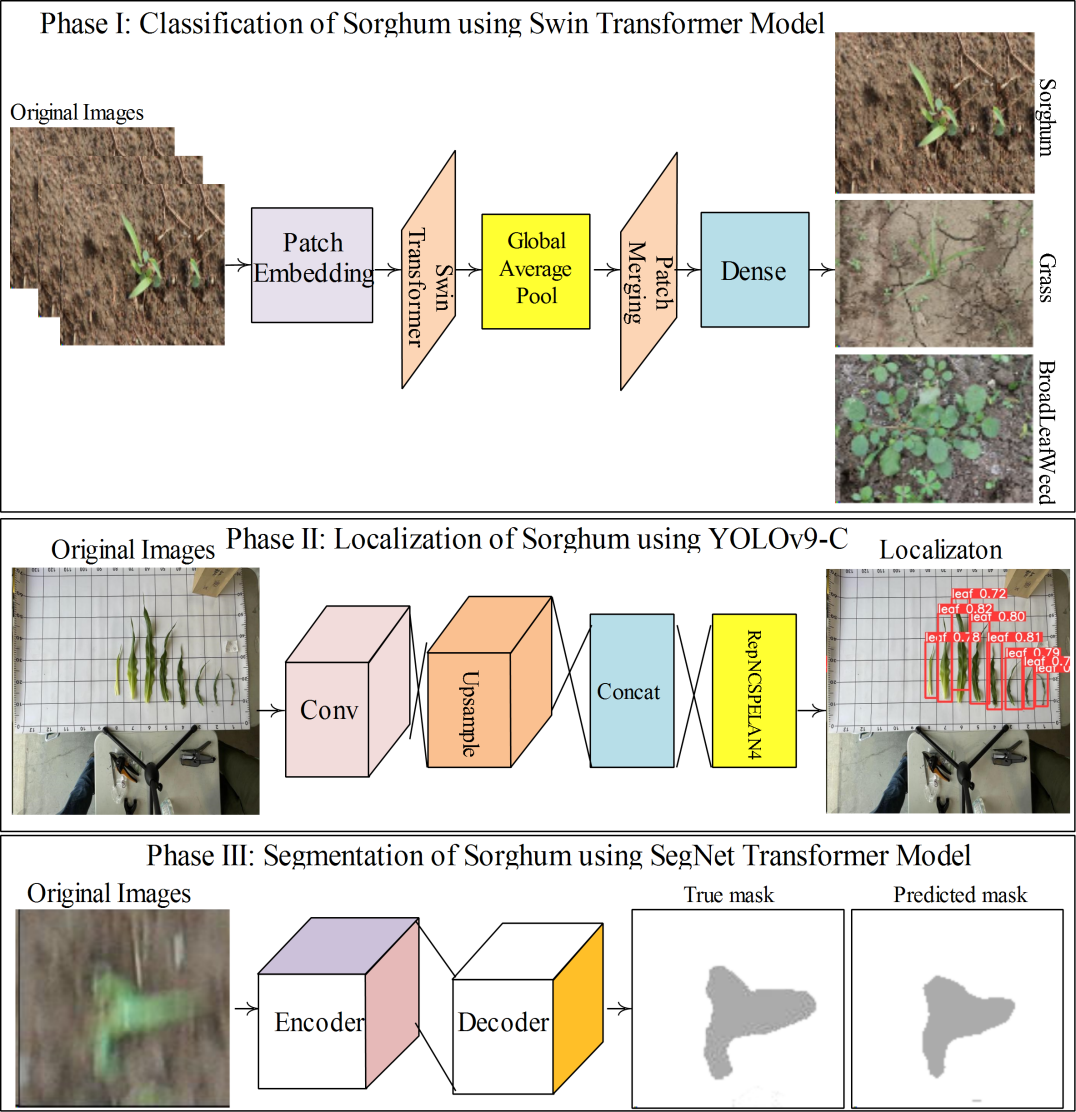
Proposed method architecture to classify, localize, and segment the sorghum leaves.

The proposed method comprises three deep-learning models: Swin Transformer (Liu et al., 2021) for classification, YOLOv9-c (Wang et al., 2024) for localization, and SegNet transformer (Badrinarayanan et al., 2017) for the segmentation of sorghum. The detailed proposed method steps are visualized in **Figure 1**.

In **Figure 1**, the proposed SWIN transformer model classifies images of sorghum, grass, and broadleaf weeds. To localize the sorghum leaf, the YOLOv9-c model is trained on hyperparameters. The proposed SegNet transformer model consists of an encoder/decoder to segment the sorghum region.

### 3.1 Classification of sorghum diseases

**Figure 2:**
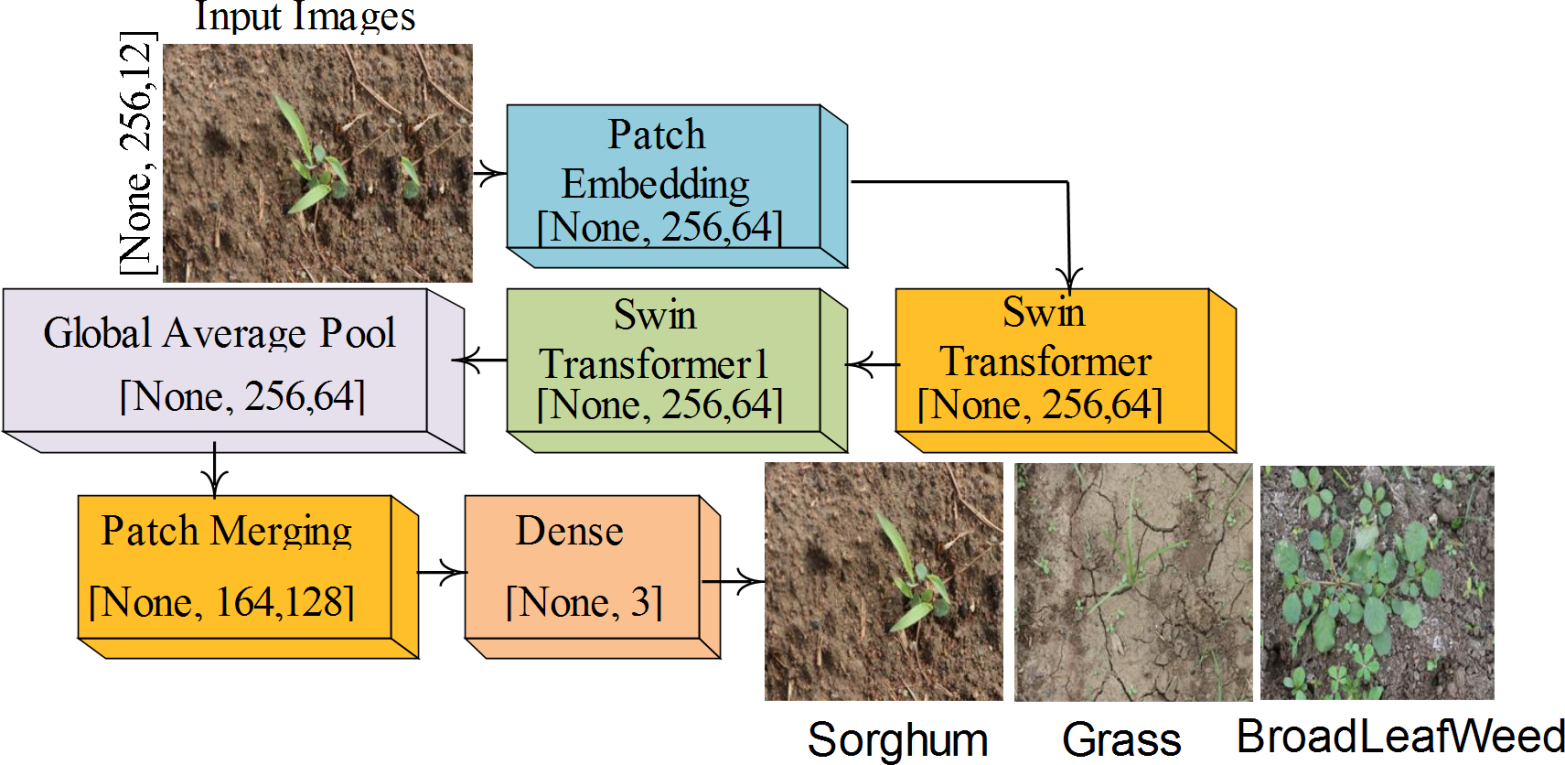
Proposed swin transformer model for classification.

The Swin Transformer (ST) is a hierarchical transformer that measures representations based on shifted windows. While allowing cross-window connections, it improves performance by restricting self-attention measurement to non-overlapping local regions. The proposed Swin Transformer consists of nine layers: input, patch embedding (PE), two Swin Transformers (ST), patch merging (PM), global average pooling (GAP), and a dense layer (DE) to classify sorghum, grass, and broadleaf weed, as shown in **Figure 2**.

The Swin Transformer model works based on a vision transformer, which focuses on localized self-attention in the region of the window and extracts features hierarchically, in which the patch embedding layer protects the data in a low-dimensional space as Equation (1).


(1) $${\text{P}}_{\text{E}}={\text{XW}}_{\text{E}}+{\text{b}}_{\text{E}}$$

Here $\text{X}\in {\text{ R}}^{\text{β}\times \text{H}\times \text{W}\times {\text{C}}_{\text{in}}}\to$ input tensor, $\text{E}\in {\text{ R}}^{\text{β}\times \text{P}\times {\text{C}}_{\text{out}}}\to$ embedded output patch, and W represents the weight. The ST model computes the attention in a non-overlapped window defined as Equation (2).


(2) $$\text{Attention}(\text{Q},\text{k},\text{ V})=\text{softmax}(\frac{\text{Q}\times {\text{K}}^{\text{T}}}{\surd {\text{d}}_{\text{k}}}+\text{b})\times \text{V}$$

Here Q represents the query matrix, K represents the key matrix, V represents the value matrix, d_k_ is the dimension of the key vectors, b is the bias term (used for attention masking or relative positional encoding), and attention weights are computed through softmax. Attention is measured individually across each window. In the multi-shifted window, the cross-window interactions were maximized, in which input patches are shifted cyclically through pixels before using window multi-scale attention. The patch output is shifted back to its original position after the attention computation. After attention, a multi-layer perceptron (MLP) is applied to fix the features across the dimensions. $\text{MLP}(\text{X})=\text{GELU}({\text{XW}}_{1}+{\text{b}}_{1}){\text{W}}_{2}+{\text{b}}_{2}$ where ${\text{W}}_{1}$ , ${\text{W}}_{2}$ denote the weights while ${\text{b}}_{1},{\text{b}}_{2}$ represents bias. Normalization is added in each layer to stabilize the model training using Equation (3).


(3) $$\text{LN}(\text{X})=\frac{\text{X}-\text{μ}}{\text{σ}+\text{ϵ}}$$

where σ,μ denote standard deviation and mean, while ϵ represents a small constant. In patch merging, a hierarchical structure is created by merging neighboring patches to reduce spatial resolution and increase feature depth given as Equation (4).


(4) $${\text{X}}_{\text{merged}}=\text{concat}({\text{X}}_{\text{P}1},{\text{ X}}_{\text{P}2},{\text{ X}}_{\text{P}3},{\text{X}}_{\text{P}4}){\text{W}}_{\text{m}}$$

${\text{X}}_{\text{P}}$ =merged patches, ${\text{W}}_{\text{m}}$ denote weights. After extracting features, global pooling collapses the spatial dimensions using Equation (5).


(5) $${\text{X}}_{\text{pooled}}=\frac{1}{\text{N}}\sum_{\text{i}=1}^{\text{N}}{\text{X}}_{\text{i}}$$

N denotes the spatial locations. The output is computed by applying a dense layer with a softmax activation using Equation (6).


(6) $$\text{Y}(\text{output})=\text{softmax}({\text{XW}}_{\text{d}}+{\text{b}}_{\text{d}})$$

Here ${\text{W}}_{\text{d}},{\text{b}}_{\text{d}}$ represents the weights and bias, while Y denotes the probabilities.

The detailed architecture of the ST model with activations is mentioned in **Table 1**.

**Table 1:** Proposed ST model architecture.

Input	Shape of output	Parameters
Input	(None, 256,12)	0
PE	(None, 256,64)	17,216
ST	(None, 256,64)	50,072
ST	(None, 256,64)	51,096
PM	(None, 64,128)	32,768
GAP	(None,128)	0
DE	(None, 3)	387

Total parameters = 151,539; trainable parameters = 150,483; non-trainable = 1,056.

The proposed model training hyperparameters are mentioned in **Table 2**.

**Table 2:** Proposed ST model hyperparameters.

Parameter	Details
Patch size	(2, 2)
Window size	2
Learning rate	1e-3
Batch size	128
Epochs	40
Weight decay	0.0001
Dropout rate	0.03
Heads	8
Embed dim	64
Mlp	256

**Table 2** provides the hyperparameters, which are finalized after extensive experiments. After classification, the classified images are passed to the YOLOv9-c for localization of the class label.

### 3.2 Localization of sorghum diseases

**Figure 3:**
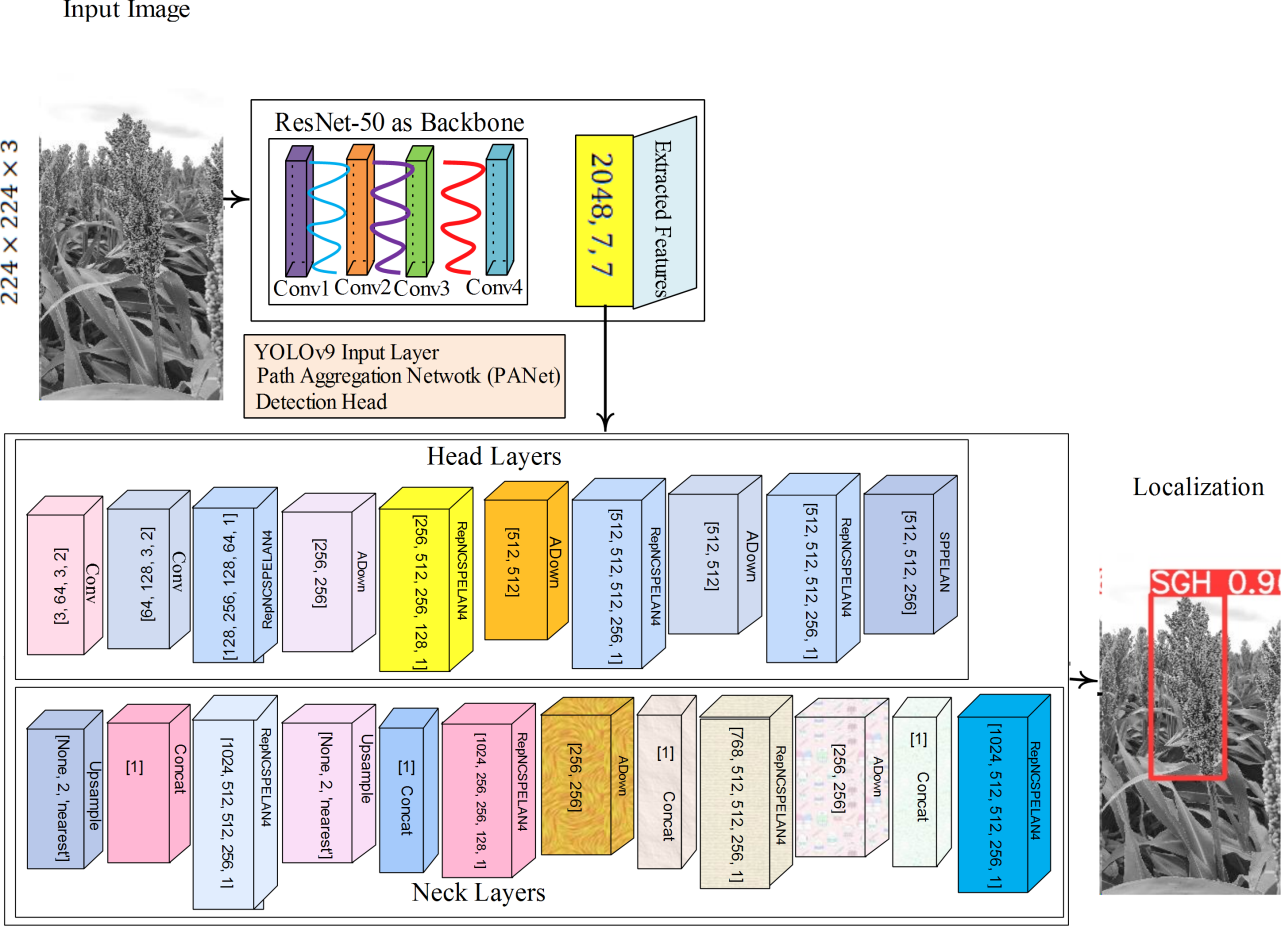
YOLOv9-c and PANet for localization/detection of the sorghum plant.

The hypothetical YOLOv9-c passes input through a neural network designed to recognize and categorize objects in real time. Convolutional layers are used in the model to extract features, and these are followed by layers that forecast box boundaries and probabilities for various objects in an image. For the YOLOv9-c model, the annotated data were prepared, training was performed, and the hyperparameters were fine-tuned to achieve optimal performance. In the training process, the images are passed to the network, and the network computes loss based on the prediction and annotated masks. Finally, the model is updated with weights based on the backpropagation algorithm. The test images are passed to the trained model to predict the correct class label with high speed and accuracy. The YOLOv9-c version is trained on sorghum images with ground-annotated masks at an eight-batch size and 100 epochs. The YOLOv9-c architecture is shown in **Figure 3**.

The ResNet50 model comprises 50 layers based on residual learning, featuring skip connections that help mitigate the problem of vanishing gradients. The initial layers of ResNet50 comprise convolutional layers (7 × 7, stride = 2 and padding = 3). The input shape is 224 × 224 × 3. After applying the convolution layer, the output is 112× 112 × 4 . This layer is used for extracting low-level features, such as textures and edges. The ReLU activation is used for non-linearity. The maxpool is applied with a window size of 3×3 , a stride of 2, and one padding. The final output shape is 56×56×64 . The second block involves residual learning, which includes four stages. Each stage consists of multiple blocks of residual that contain a convolution with a 1×1 filter to reduce the dimension. A convolution with a 3 × 3 filter size is applied for feature extraction. A 1 × 1 convolution was used again to expand the convolution. Finally, skip connection is used to add input to improve/enhance the flow of the gradient. In stage 1, 21 to 23 layers are included, with an input size of 56×56× 64. Three residual blocks, each with filter sizes of 1 × 1, 3 × 3, and 1 × 1, respectively, had 64 and 256 channels . The output is 56×56×256 . In stage 2, 31 to 34 layers are included; at this stage, the input size is 56 × 56 × 256 . The four residual blocks have filter sizes of 1×1, 128, 3×3, 128, 1×1, 512 . The final output shape is 28×28×512. . In stage 3, the input shape of 28 × 28 × 512 is passed to the six residual blocks. Each block has a filter size of 1×1, 3×3, and 1×1, with a 1024 output shape, resulting in a 14×14×1024 output. In stage 4, the input size is 14 × 14 × 1,024, comprising three residual blocks, each containing filters of 1 × 1, 3 × 3, and 1 × 1, with dimensions of 512, 512, and 2,048, respectively. The final output shape is 7×7×2048. The global average pool layer is 7 × 7 × 2048. The final layer dimension of 7×7×2048 is converted into a 1D vector of size 2,048, mapping 2,048 features to the number of classes.

#### 3.2.1 Extracted features using YOLOv9

The extracted features output to the last block of the convolutional (stage 4) of Resnet50 is applied. The 7 × 7 × 2,048 feature shape is fed to YOLOv9-c, which processes these features using a path aggregation network (PANet) for multi-scale feature fusion. The detection head generates the coordinates of the bounding box and class probabilities. ResNet50 used sequential convolutional layers. ${\text{F}}_{\text{resnet}}={{\text{f}}^{\left(\text{l}\right)}}_{\left(\text{X}\right)}={\text{w}}^{\left(\text{l}\right)}\times \text{X}+{\text{b}}^{\left(\text{l}\right)}$ where X is the input, ${\text{w}}^{\left(\text{l}\right)}$ weight, ${\text{b}}^{\left(\text{l}\right)}$ bias of lth convolutional layer. ${\text{F}}_{\text{resnet}}={\text{f}}_{\text{conv}4}({\text{F}}_{\text{Conv}3})$ , where Conv3 and Conv4 denote the features from layers 3 and 4, respectively. The size of the features from layer 4 is  (2048× 7, 7) as an input size of 224 × 224.

### 3.2.2 YOLOv9-PANet aggregation

The features map ${\text{F}}_{\text{resnet}}$ are processed by YOLOv9-c path aggregation network (PANet) ${\text{F}}_{\text{PAN}}\text{Concat}[{\text{f}}_{\text{up}}({\text{F}}_{\text{resnet}}),{\text{ f}}_{\text{down}}({\text{F}}_{\text{resnet}})]$ , where ${\text{f}}_{\text{up}}$ and ${\text{f}}_{\text{down}}$ denote the features from up and down, respectively. YOLOv9-c utilizes anchors for predicting bounding boxes. ${\text{B}}_{\text{prediction}}$ = σ(x) .w+b. The SoftMax activation is applied to predict the class labels. ${\text{P}}_{\text{class}}(\text{c})=\frac{{\text{e}}^{\text{Zc}}}{\sum_{\text{j}=1}^{\text{c}}{\text{e}}^{\text{Zj}}}$ where Zc denotes (c) class logits. The default hyp.scratch-high.yaml is used from the YOLOv9-c repository, and critical training hyperparameters are given in **Table 3**.

**Table 3:** Hyperparameters of the localization model.

Parameter	Value
lr0	0.01
Lrf	0.1
Momentum	0.937
Weight decay	0.0005
Warmup epochs	3.0
Box	0.05
Cls	0.5
Obj	1.0
Label smoothing	0.0
hsv-h, hsv-s, hsv-v	0.015, 0.7, 0.4
Degrees, translate, scale, shear	0.0, 0.1, 0.5, 0.0

These hyperparameters are chosen for high-accuracy optimization on agricultural datasets and are editable for different tasks.

YOLOv9-c configuration file (yolov9-c.yaml) is utilized, which is designed for compact and efficient object detection with high accuracy. This variant includes the following components:

Backbone: CSPRep3-based feature extractor with SPPF; neck: PAN-FPN structure for multi-scale feature fusion; head: decoupled head for classification and localization; attention: includes hybrid task cascade module with EMA and DFL. This architecture strikes a balance between model complexity and speed, optimizing it for real-time plant and weed detection tasks in smart agriculture.

The dataset is defined in the data.yaml file. The data is split into three files: training set (70% of the total labeled data), validation set (15%), and testing set (15%). All sets are stratified to ensure balanced class representation. We ensured no data leakage between sets by random shuffling with a fixed seed before splitting.

The summary of the training parameters is given in **Table 4**.

**Table 4:** Training parameters of the localization model.

Workers	4
Device	0
Batch	8
Epochs	100
img 640	Input image resolution
cfg yolov9-c.yaml	compact model configuration
Weights	Training from scratch

These details confirm that the model training procedure and experimental settings are rigorously defined and reproducible. These specifications are explicitly included in the revised manuscript for clarity and transparency.

### 3.3 Segmentation of sorghum diseases

The vision transformer models performed better on tasks compared to classical CNN, such as semantic segmentation. The transformers model, based on self-attention, considers the entire context of the image, which provides more information to capture global dependencies. In contrast, CNN relies only on local information based on convolution. The Segformer model is applied in conjunction with the U-Net model for segmentation, where features are extracted using Segformer. The process includes spatial encoding and channel information of the input data. $\text{I}={\text{R}}^{\text{H}\times \text{W}\times \text{C}}$ where ${\text{I}}_{\text{H}}$ is height, ${\text{I}}_{\text{W}}$ is weight, and C is the channel of the input images. The segformer model processes the input images into patch embedding and sends them to the multi-head attention layers. The input encoding process is $\text{O}=\text{transformer }({\text{I}}_{\text{patches}})$ , where O $\epsilon {\text{R}}^{\text{P}\times \text{D}}$ , O is the output embedding, P represents patches, and D denotes the embedding dimension. The input image size is 128×128 , and the patch size is 16. Number of patches O = $(\frac{{\text{I}}_{\text{H}}}{\text{P}})\times (\frac{{\text{I}}_{\text{W}}}{\text{P}})=(\frac{128}{16})\times (\frac{128}{16})=8\times 8=64$ . The images are divided into a total of 64 patches, and the dimensions of the patches are D = P×P=64×64=256 pixels (element). The multi-head attention is applied to capture different patterns or dependencies through multiple heads of attention, which focus on distinct parts of the input in parallel. Each head has input independently, which permits the model to attend to different contents and features in the data.

**Figure 4:**
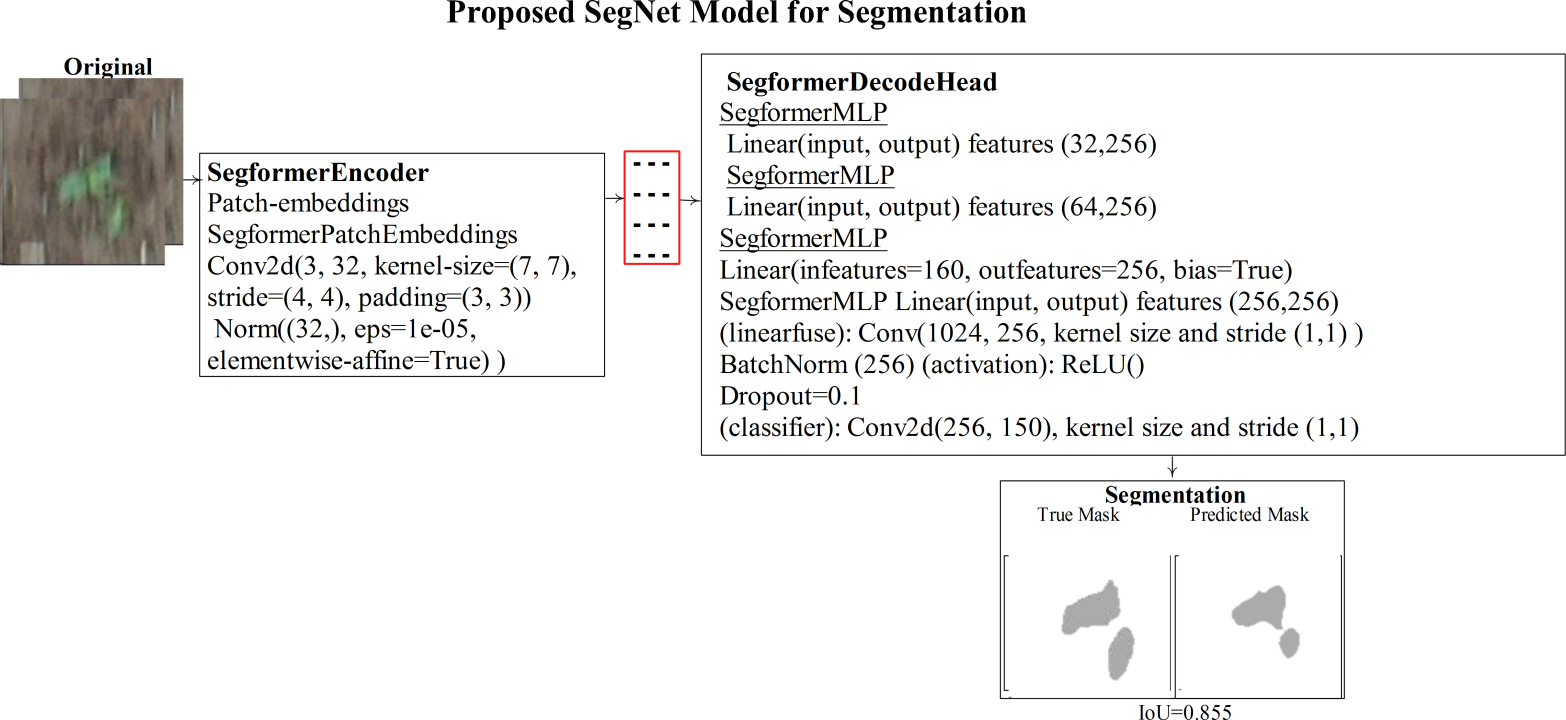
Segmentation of sorghum using the SegNet transformer model.

O'=softmax(QKTdK)V (7).

where Q is query, K is key, V is value, and ${\text{d}}_{\text{k}}$ is a vector dimension. $\text{Q}={\text{Om}}_{\text{Q}},\text{ K}={\text{Om}}_{\text{K}},\text{ V}={\text{Om}}_{\text{V}}$ , The ${\text{m}}_{\text{Q}},\;{\text{m}}_{\text{K}},{\text{m}}_{\text{V}}\;$ denotes the linear matrices to project O into the query, key, and values spaces. The number of columns in these matrices is the same as the O. The shape of these matrices is ${\text{m}}_{\text{Q}},\;{\text{m}}_{\text{K}},{\text{m}}_{\text{V}}=(256,{\text{d}}_{\text{K}}).$ The O is multiplied by the ${\text{m}}_{\text{Q}},\;{\text{m}}_{\text{K}},{\text{m}}_{\text{V}}$ then Q, K, V: (64, ${\text{d}}_{\text{K}}$ ) = (is computed attention), $\frac{{\text{QK}}^{\text{T}}}{\sqrt{{\text{d}}_{\text{t}}}}$ compute the scalar dot product among the query and key matrices. The ${\text{QK}}^{\text{T}}$ shape is as: Q: (64,256), ${\text{K}}^{\text{T}}:(256,64)$ and the final result ${\text{QK}}^{\text{T}}=(64,64).$ . The matrix size is (64, 64), which denotes the attention scores between every pair of patches. Then, softmax is applied to the (64, 64) matrix to normalize the attention scores and convert them into probabilities. The output is computed by multiplying the attention scores by the V matrix value. The output shape is attention scores (64, 64) V = (64,256), ${\text{O}}^{'}=(64,\;256)$ having the same shape as the original input O. The extracted features from the segformer model are passed to the decoder module, and the output of the features is concatenated, which is achieved from different transformer stages ${\text{F}}_{\text{concatenated}}=\text{concat }({\text{F}}_{1},{\text{ F}}_{2}\ldots .{\text{F}}_{\text{N}})$ . The cross-entropy loss is computed between the predicted mask ${\text{y}}_{\text{pred}}$ and the actual mask ${\text{y}}_{\text{actual}}$ ${\text{E}}_{\text{L}}({\text{y}}_{\text{pred}},{\text{ y}}_{\text{actual}})=\sum_{\text{i}=1}^{\text{N}}\sum_{\text{c}=1}^{\text{C}}{\text{y}}_{\text{true }(\text{i},\text{c})}$ log ${\text{y}}_{\text{pred }(\text{i},\text{c})}$ , where N represents the number of pixels, C is the number of classes, ${\text{y}}_{\text{pred }(\text{i},\text{c})}$ denote the predicted probability of class c for pixel I. Then, the model is trained using the Adam optimizer with a learning rate of lr = 5e-5 and a batch size of 8. The architecture of the SegNet model is shown in **Figure 4**.

Therefore, the SegNet transformer model is proposed for sorghum segmentation. The sorghum region is segmented using the pre-trained segformer-b0-finetuned-ade-512-512 (Xie et al., 2021) model that is trained on sorghum images with ground masks and selected hyperparameters such as 10 epochs, 8 batch size, Adam, lr = 5e-5, and CrossEntropyLoss.

## 4 Results and discussion

The classification dataset of sorghum weeds contains 4,312 images to address the problem of crop weeds. The segmentation dataset of sorghum weeds comprises 5,555 manually annotated segments from 252 samples, addressing the problem of segmentation (Justina and Thenmozhi, 2024b). Five sorghum localization datasets are used. The sorghum detection dataset contains only one class of sorghum and a total of 126 images, comprising 88 training, 24 validation, and 14 testing images (Song, 2022). The SGH localization dataset comprises 748 images prepared by Kansas State University, with 420 images for training, 40 for validation, and 20 for testing (K. S. University, 2023). The sorghum leaf localization dataset comprises 1,192 images, of which 982 are used for training, 70 for testing, and 140 for validation (K. C. Lab, 2023). The dataset contains 147 panicles of sorghum and counts of grain (James et al., 2024). The sorghum grain head dataset contains three folders: training, testing, and validation, in which 1,500 images are for training, 102 for validation, and 21 for test images (K. S. University, 2022).

To support the robustness of the proposed method, the dataset descriptions include detailed statistics, such as class distributions, image resolutions, and environmental variability. These datasets reflect diverse real-world conditions such as varying lighting, occlusions, and backgrounds, making them suitable for training resilient deep learning models. A detailed summary of the datasets used is provided in **Table 5**.

**Table 5:** Description of datasets.

Dataset	Total images	Split (training/validation/testing)	Classes	Resolution/quality	Environmental variability
Sorghum weed classification	4,312	4,312/862/431	3 (sorghum, grass, broadleaf weed)	Various (approximately 640 × 640)	Field lighting, natural variations
Sorghum weed segmentation	5,555 segments from 252 images	202/25/25	Pixel-wise annotated	High resolution	Manual segmentation under field conditions
Sorghum detection (single class)	126	88/24/14	1 (sorghum)	Mixed resolution	Natural background, illumination changes
SGH Localization (Kansas State)	748	420/40/20	1 (sorghum head)	Standard resolution	The field was captured under different sunlight conditions
Sorghum leaf localization	1,192	982/140/70	1 (leaf)	High resolution	Various lighting and occlusion scenarios

The proposed models for classification, localization, and segmentation are executed on an NVIDIA GeForce RTX 4060 Ti GPU (16 GB VRAM), utilizing approximately 2.6 GB of memory with an average GPU utilization of 62%. These observations confirm the computational efficiency of the models. The current implementation demonstrates fast processing speeds, approximately 2.1 ms/image for classification, 7.8 ms/image for YOLOv9-c localization, and 13.5 ms/image for transformer-based segmentation. The real-time deployment and optimization on edge or embedded systems are planned for future work to further validate performance under operational field conditions.

### 4.1 Experiment #1: classification of sorghum

**Figure 5:**
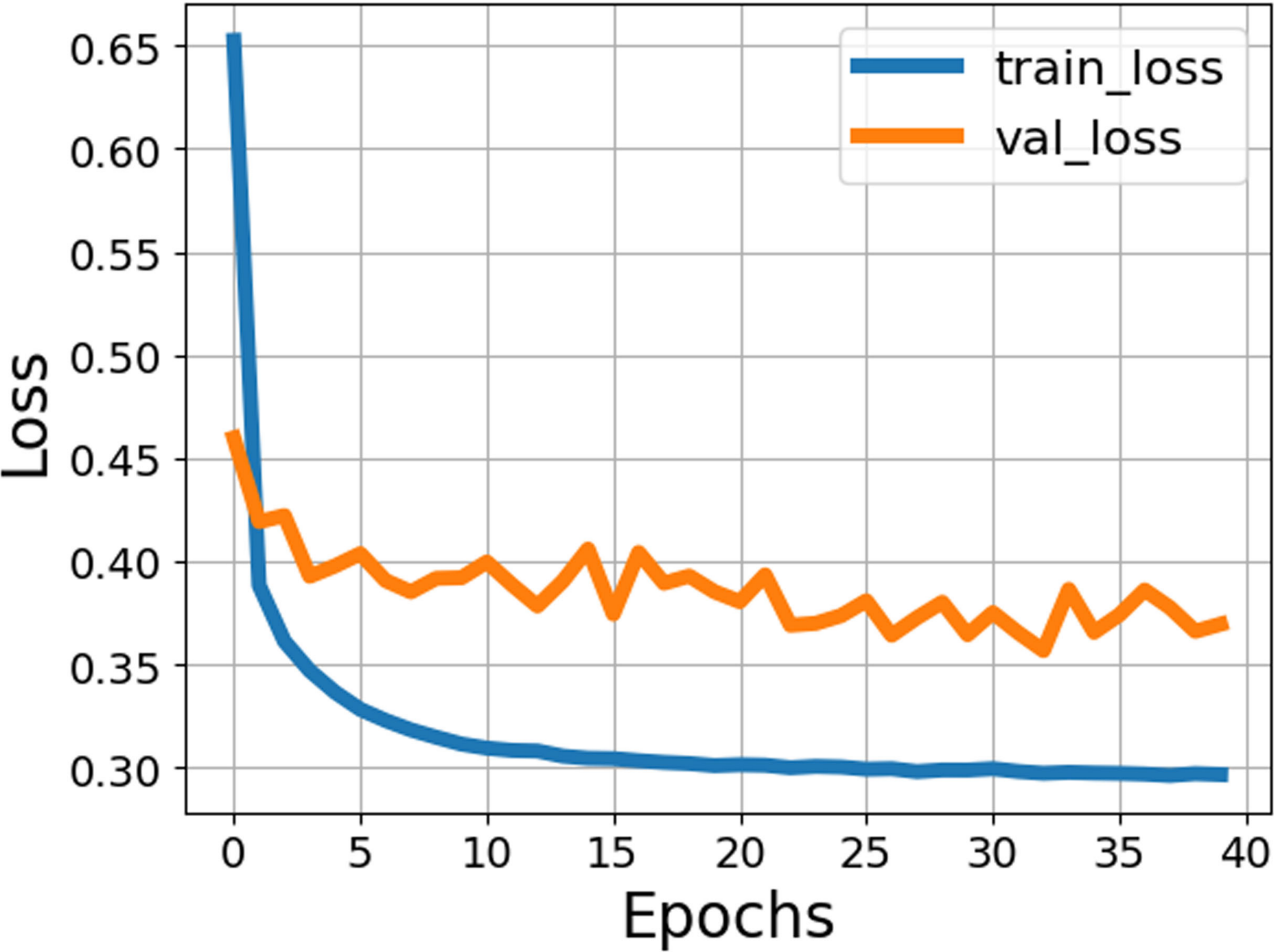
Training performance of the classification model.

The proposed ST model classified the data into sorghum, grass, and broadleafweed. The model is trained for 40 epochs, and the loss rates for training and validation are plotted in **Figure 5**.

In the graph, on 40 epochs, the train loss is 0.30, and the validation loss is less than 0.40.

The sorghum weed classification dataset contains three subfolders: train, valid, and test, in which each folder has three classes, such as sorghum, grass, and broadleaf weed. The training data contains 1,404, 1,467, and 1,441 images of the sorghum, grass, and broadleaf weed classes, respectively. The validation data consists of 281, 293, and 288 images for the three classes, respectively. The testing data contains 140, 147, and 144 images of three classes.

For classification, in this research, the folders of train and validation data are combined to create a single folder. The train folder contains 1,404 + 1,467 + 1,441 = 4,312 images, and the validation folder contains 281 + 293 + 288 = 862 images. After combining the train and validation folders, the total number of images is 5,174. The test folder contains 140 + 147 + 144 = 431 images. The augmentation methods are applied in terms of vertical/horizontal flipping, rotation, scaling, etc., to increase the number of images. After augmentation, the total number of training images is 36,635, and the test images are 3,003. The two separate folders, train and test, are passed to the proposed classification model for training, and model performance is evaluated on the 3,003 testing data.

**Figure 6:**
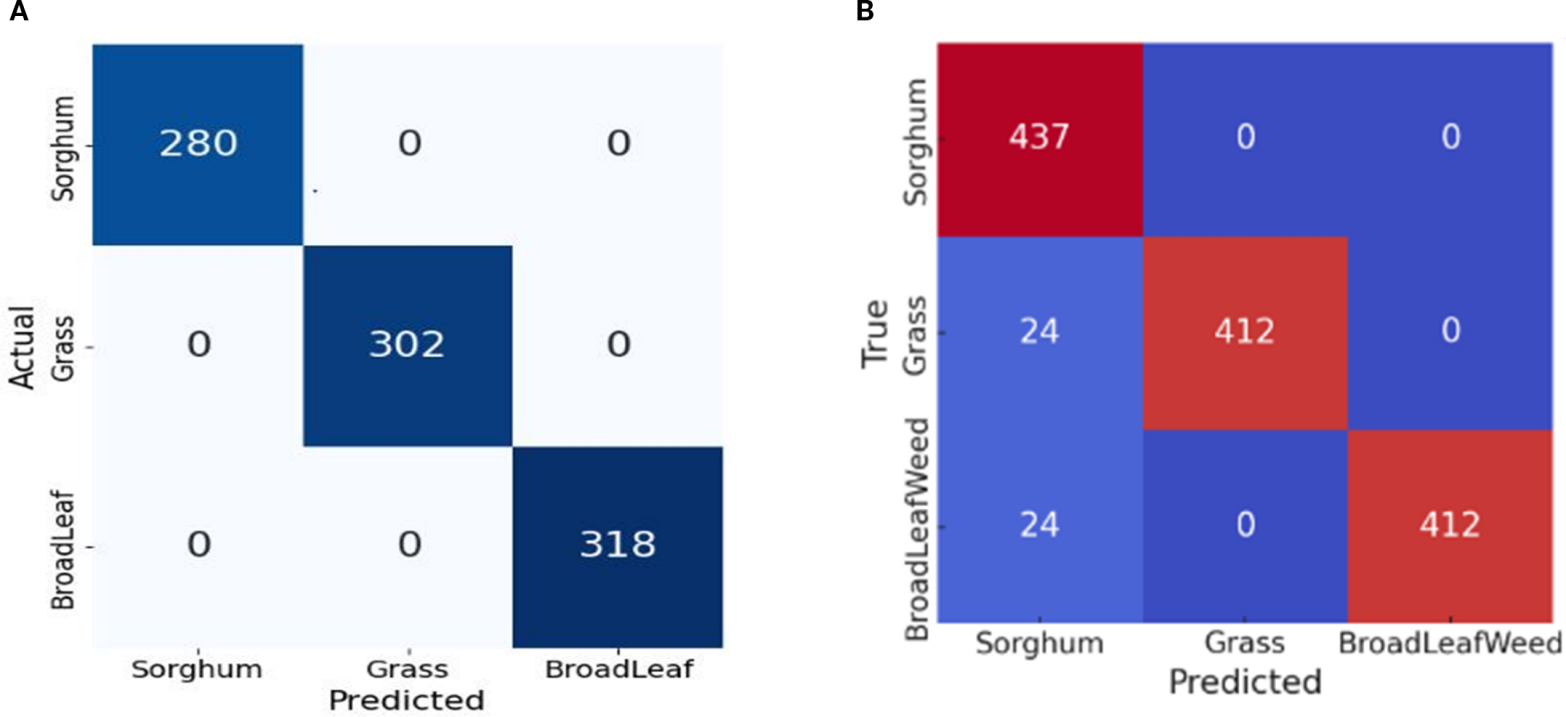
Confusion matrix of classification model. **(A)** With augmentation. **(B)** Without Augmentation.

Similarly, the classification results are computed, where the entire training and test data are combined into a single folder and split into training and testing sets using a 0.2 holdout validation. The confusion matrix based on model performance is visualized in **Figure 6**.

The performance metrics are computed using the confusion matrix, which is provided in **Table 6**.

**Table 6:** Classification results with and without augmentation.

Augmentation Status	Sorghum	Grass	Broadleaf weed	Precision	Recall	F1-score	Overall accuracy
Without augmentation	☑			0.90	1.00	0.94	0.96
		☑		1.00	0.94	0.97	
			☑	1.00	0.94	0.97	
With augmentation	☑			1.00	1.00	1.00	1.00
		☑		1.00	1.00	1.00	
			☑	1.00	1.00	1.00	

In **Table 6**, for the class of sorghum, 0.90 precision, 1.00 recall, and 0.94 F1-score are achieved. In the grass class, a precision of 1.00, a recall of 0.94, and an F1-score of 0.97 are obtained. Similarly, on the broadleaf weed class, a precision of 1.00, a recall of 0.94, and an F1-score of 0.97 are achieved. The overall accuracy in the three classes is 0.96. After augmentation, the data is balanced in each class, which increases the classification results. The complex statistical analysis of the classification model is given in **Table 7**.

**Table 7:** Statistical analysis of the classification model.

Metric	Value
Matthews correlation coefficient (MCC)	0.946
Log-loss/cross-entropy loss	0.070
McNemar’s test (vs. perfect baseline)	*p*-value ≈ 9.76 × 10^-^²^6^
McNemar contingency table	[[2,891, 0], [112, 0]]

The statistical results based on the confusion matrix show an overall accuracy of 96.3%. The 95% confidence interval for accuracy is [95.59%, 96.95%], and the chi-square test *p*-value is <0.0001. Cohen’s kappa was 0.944, indicating an excellent agreement beyond chance. Matthews correlation coefficient (MCC) was 0.946, indicating high model reliability across all classes. These results demonstrate the model’s strong predictive performance while also confirming (via McNemar’s test) that it performs statistically differently than a perfect classifier.

**Figure 7:**
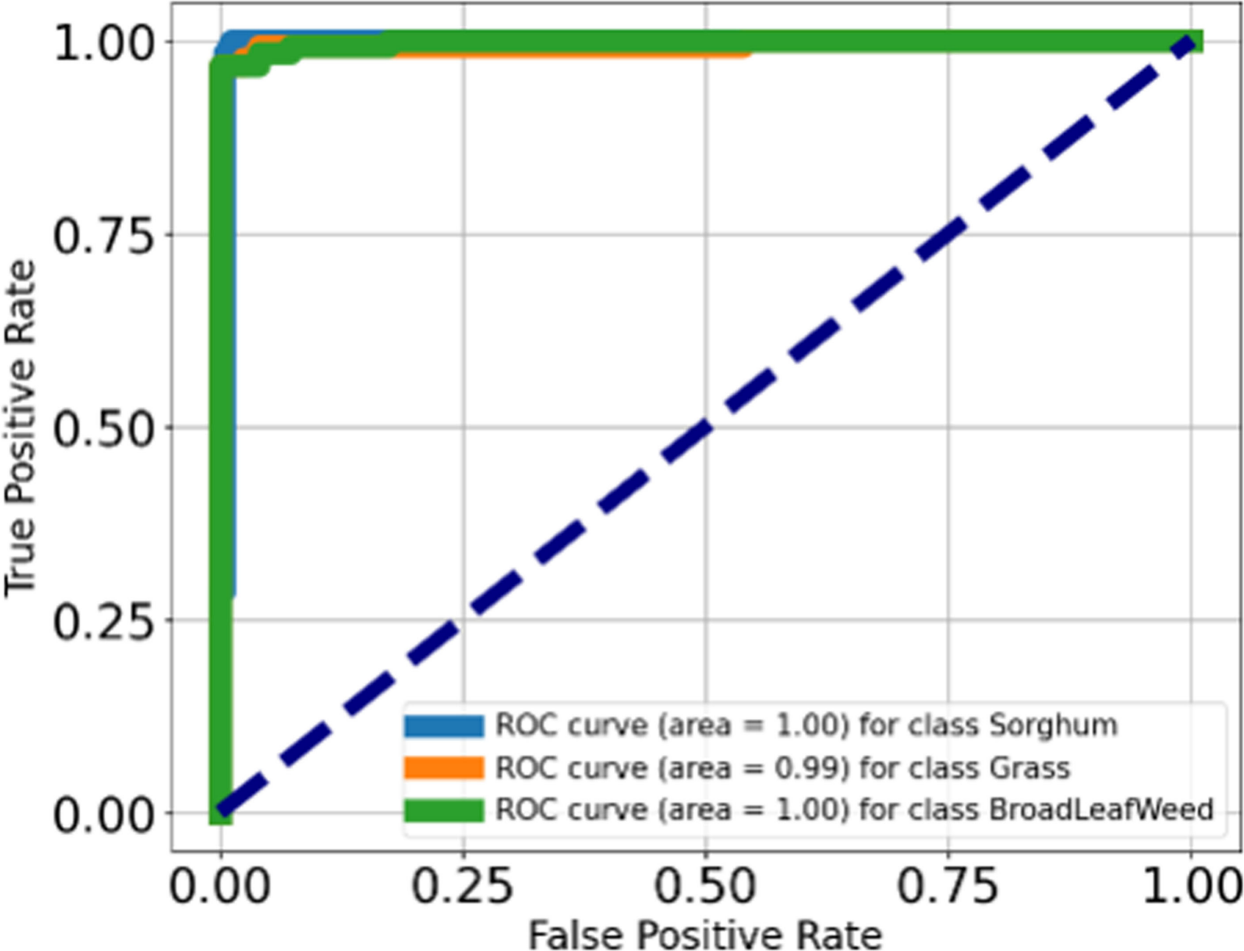
ROC of the proposed classification model.

ROC is also computed on each class separately and plotted in **Figure 7**.

In **Figure 7**, the AUC classes of 1.00 on sorghum, 0.99 on grass, and 1.00 on broadleafweed are shown. **Table 8** provides a comparison of the achieved results.

**Table 8:** Comparison of classification model results with the existing techniques.

Reference	Year	Dataset	Accuracy, %
(Park et al., 2023)	2023	Classification dataset of sorghum weeds	91.0
(Justina and Thenmozhi, 2024a)	2024		98.6
(Gonzalez et al., 2024)	2024		86.9
(Sandosh et al., 2025)	2025		99.0
Proposed model			1.00

In **Table 8**, the U-net model is applied on RGB sorghum images for detection with an accuracy of 91.0 (Park et al., 2023). HierbaNetV1 model is used, which consists of 72 layers for the classification of the sorghum weeds with 98.6% accuracy (Justina and Thenmozhi, 2024a). The fully convolutional (FCN) and EfficientNet-B3 networks are employed for the detection of sorghum weeds. FCN gives better results compared to EfficientNet-B3, with accuracy of 86.97% and 97.76%, respectively (Gonzalez et al., 2024). The DenseNet-169 model’s features are fine-tuned for classification, and the optimal features are visualized using the LIME and GradCam methods (Sandosh et al., 2025). However, compared to the existing model, the ST model is proposed and trained from scratch on optimal layers and hyperparameters, which provide better outcomes.

### 4.2 Experiment #2: localization of sorghum

In this experiment, the localized sorghum region using the YOLOv9-c model is trained on 100 epochs and 8 batch size. The localization outcomes are computed in terms of recall, precision, and mAP50 on four benchmark sorghum datasets, as listed in **Table 9**.

**Table 9:** Localization results on the benchmark datasets.

Datasets	*P*	*R*	mAP50
SGH	1.00	0.995	0.996
Sorghum leaf detection	0.980	0.976	0.982
Sorghum panicles	0.931	0.980	0.961
Sorghum grain head	1.00	0.960	0.898

**Figure 8:**
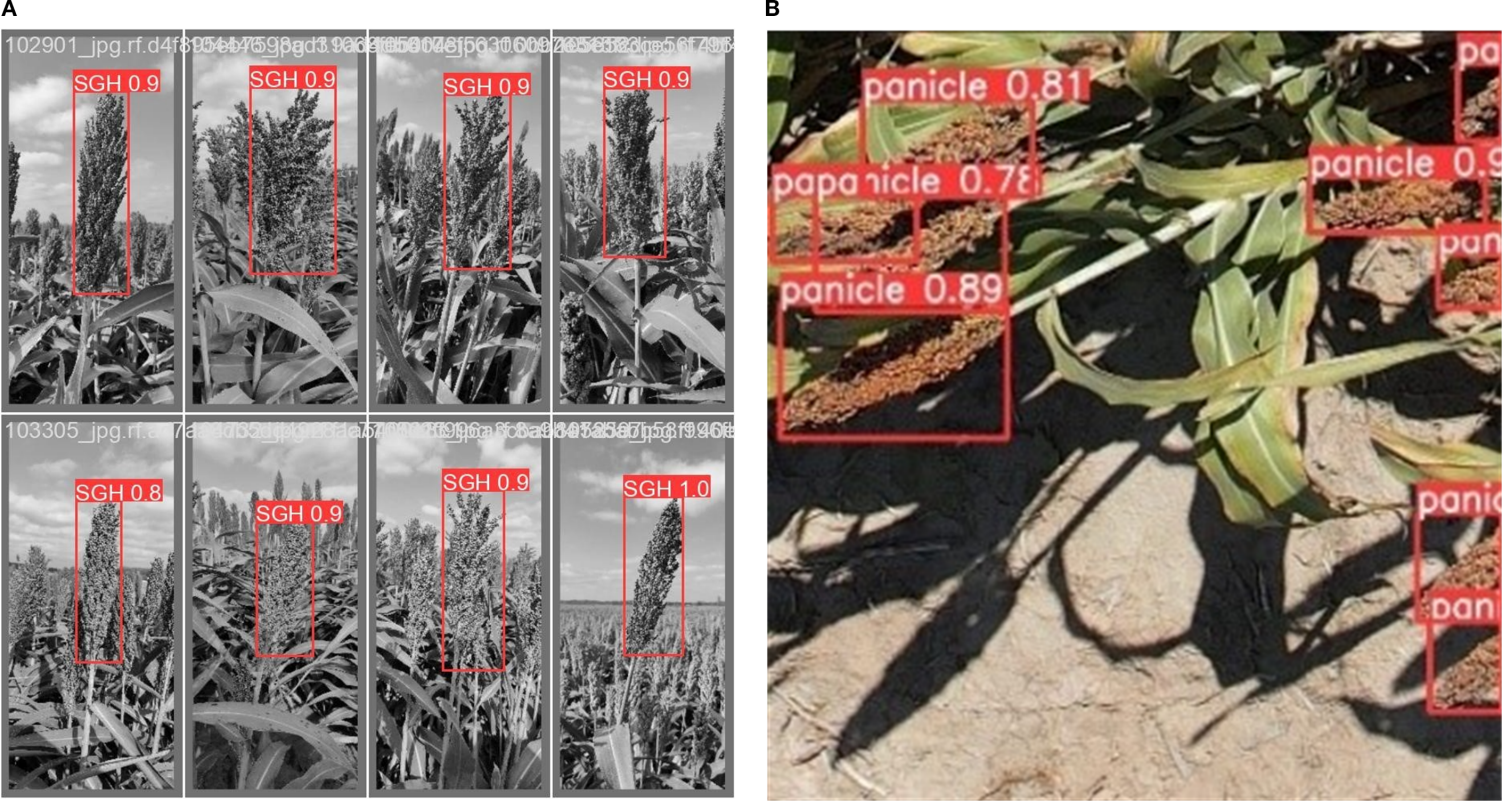
Localization results on the benchmark datasets. **(A)** SGH and **(B)** sorghum panicle.

**Table 9** provides the localization outcomes on the SGH and sorghum leaf detection datasets. The achieved results on the SGH dataset were 1.00 precision, 0.995 recall, and 0.996 mAP50. On the sorghum leaf detection dataset, the results are 0.980 precision, 0.976 recall, and 0.982 mAP50. Then, 0.931 precision, 0.980 recall, and 0.961 mAP50 are achieved on the sorghum panicles dataset. Similarly, 1.00 precision, 0.960 recall, and 0.898 mAP50 are obtained on the sorghum grain head dataset. The proposed method more accurately localized the sorghum and the sorghum leaf. The proposed model localized the sorghum grain head with the highest mean average precision (mAP), as shown in **Figure 8**.

**Figure 9:**
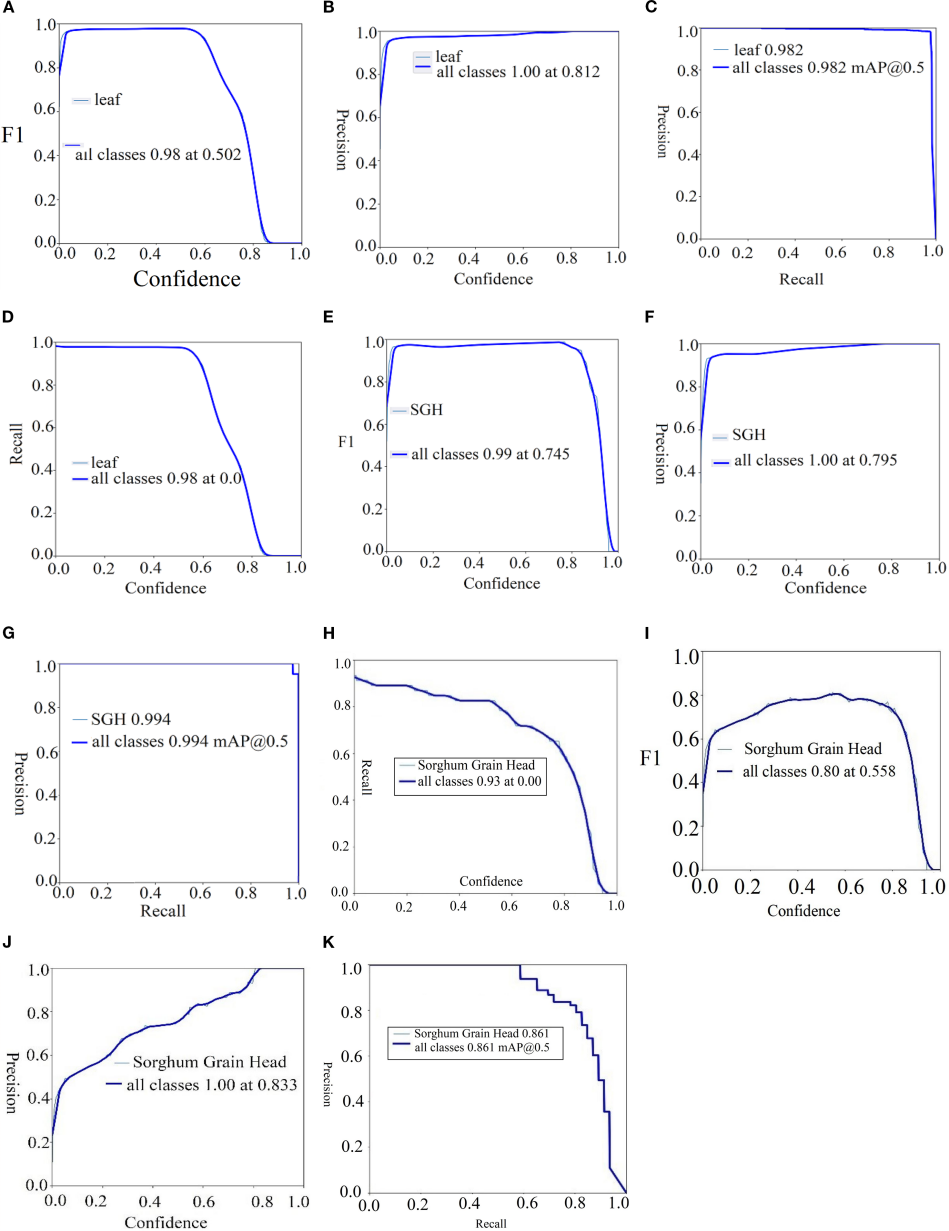
Localization results of sorghum in terms of F1-score, precision, recall, and mAp. **(A–D)** Sorghum leaf, **(E–G)** SGH, and **(H–K)** sorghum grain head.

The visualization results in **Figure 8** depict that the required region is localized with the highest prediction scores on the SGH and sorghum leaf detection datasets. The achieved results are graphically plotted in **Figure 9**.

The comparison of results is depicted in **Table 10**.

**Table 10:** Results of localization compared to existing methods.

Reference	Year	Datasets	Models	mAP
(K. S. University, 2022)	2022	Sorghum grain head	YOLOv8	0.426
**Proposed model**			YOLOv9-c	**0.898**
(James et al., 2024)	2024	Sorghum panicle	YOLOv5	0.955
**Proposed model**			YOLOv9-c	**0.961**
(K. S. University, 2023)	2023	SGH	YOLOv8	0.995
**Proposed model**			YOLOv9-c	**0.996**

The bold text represents the results of the proposed method.

The CNN model is applied to localize the sorghum grain head, achieving 0.426 mAP (K. S. University, 2022). YOLOv5 is applied to localize the panicle of the sorghum that provided mAP of 0.955 (James et al., 2024). The model is designed for localization and obtained 0.995 mAP (K. S. University, 2023). In comparison to existing works, the YOLOv9-c model is applied for localization using the selected hyperparameters, which yields the highest mAP scores among others.

### 4.3 Experiment #3: segmentation of sorghum

**Figure 10:**
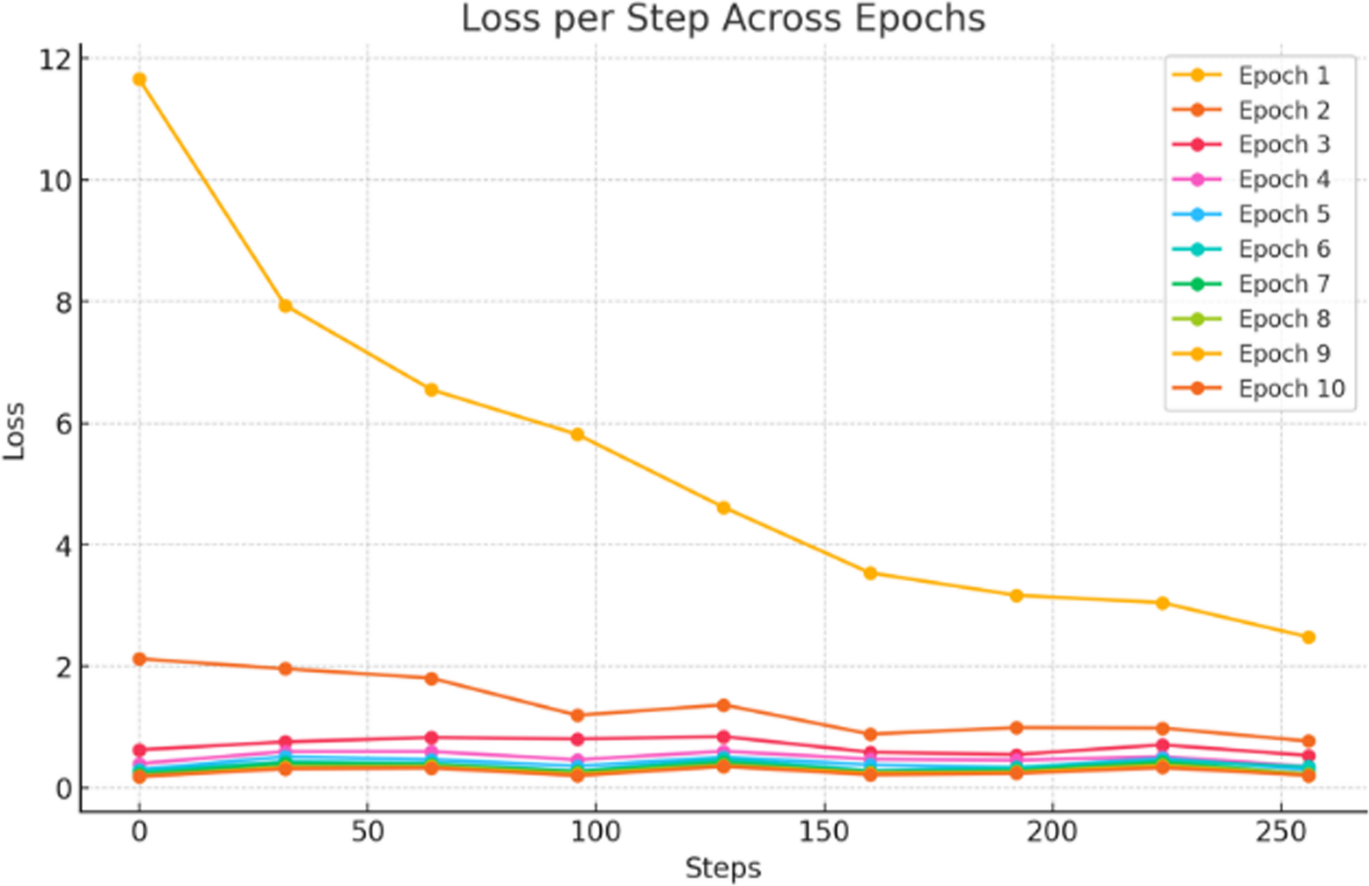
Training of the proposed Segformer transformer model for sorghum segmentation.

The segNet transformer model is trained on 10 epochs, and the training loss rate on each step across epochs is shown in **Figure 10**.

The loss rate is computed across 10 epochs, each of which is completed in 50 steps, and the 10 epochs are completed in a total of 500 steps. After each epoch, the loss rate decreased, as given in **Figure 10**. Sorghum is segmented using the proposed segmentation model. The segmentation results are computed in terms of intersection over union (IoU), and dice scores (DS) are given in **Table 11**.

**Table 11:** Segmentation results on the sorghum weeds dataset using the SegNet model.

Reference	Datasets	Model	IoU	DS
(Genze et al., 2023)	Segmentation dataset of sorghum weeds	MSEA-Net	–	0.8373
(Syed et al., 2025)		UNet	0.87	
Proposed model		SegNet	0.8973	0.9459

**Table 11** presents a comparison of segmentation results with an existing method. ResNet-50 is used as an encoder and U-net decoder for segmentation of the sorghum weeds with a DS of 0.8373. Compared to the proposed SegNet model, which achieves an IoU of 0.8973 and a DS of 0.9459, these results are significantly better.

**Figure 11:**
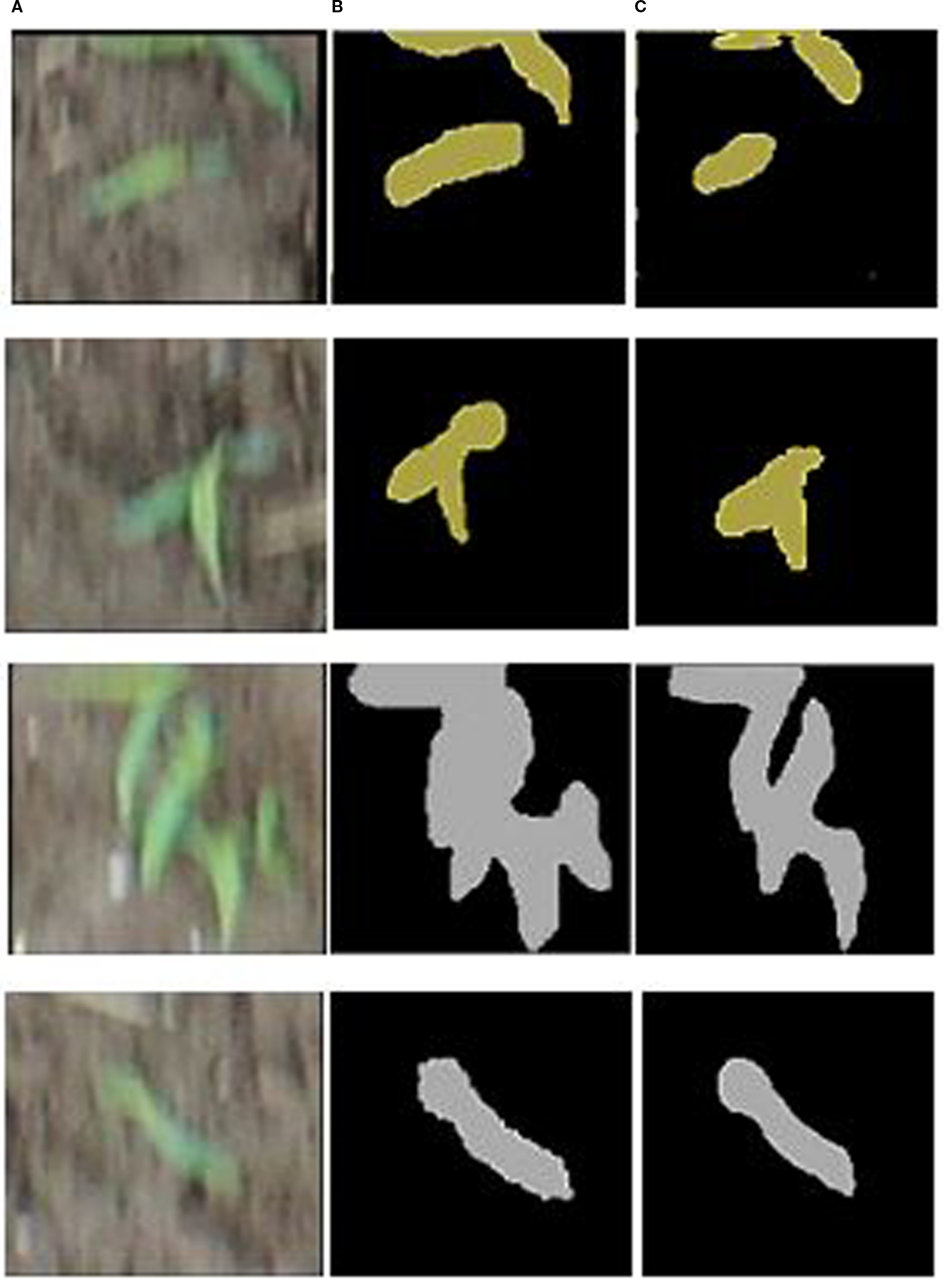
Sorghum segmentation results. **(A)** Input images, **(B)** true masks, and **(C)** predicted masks.

A lightweight MSEA-Net model is designed for segmenting sorghum weeds, achieving an IoU of 0.8742 (Syed et al., 2025). The proposed segmentation model yields prediction and ground truth masks, which are illustrated in **Figure 11**.

In **Figure 11**, the proposed segmentation model more accurately segments the sorghum. In the visualization outcomes, the predicted mask is approximately close to the true mask.

### 4.4 Ablation study

The ablation study was conducted to select optimal hyperparameters of the classification model, as listed in **Table 12**.

**Table 12:** Ablation study for the selection of optimal hyperparameters.

Embed dim	Heads	Dropout rate	Testing accuracy
**64**	**8**	**0.03**	**1.00**
32	8	0.01	0.965
64	4	0.03	0.973
32	4	0.03	0.960
64	8	0.01	0.960
32	4	0.01	0.949

The bold text indicates the selected hyperparameters that are utilized for model training.

In **Table 12**, the experimental results highlight the critical influence of hyperparameter selection on model performance. Among the various configurations tested, the combination of embedding dimension = 64, attention heads = 8, and dropout rate = 0.03 yielded the highest testing accuracy of 1.00, indicating its effectiveness for this specific classification task. However, the model’s sensitivity to changes in these hyperparameters poses a notable limitation. Small variations in embedding dimension, number of heads, or dropout rate resulted in considerable performance drops (e.g., accuracy decreasing to 0.949), underscoring the model’s reliance on precise tuning.

Similarly, the ablation study is carried out to finalize the localization and segmentation models as given in **Table 13**. In the localization model, experiments are performed on a combination of hyperparameters, including learning rate, weight decay, image resolution, and object loss weight. The model performance is evaluated using the mAP@0.5 metric.

**Table 13:** Ablation study for YOLOv9-c object detector.

lr0	Momentum	Weight decay	Image size	Obj loss weight	mAP@0.5
*0.01*	*0.937*	*0.0005*	*640*	*1.0*	*0.915*
0.005	0.937	0.0005	640	1.0	0.892
0.01	0.85	0.0005	640	1.0	0.873
0.01	0.937	0.001	640	1.0	0.901
0.01	0.937	0.0005	512	1.0	0.881
0.01	0.937	0.0005	640	0.7	0.888

In **Table 13**, the selected hyperparameters, highlighted in bold and italic, yield the highest mAP score of 0.915 compared to the others. The transformer-based SegNet model (SegFormer-B0) is evaluated using different learning rates, optimizers, and loss functions, and its performance is assessed using the Dice score and IoU, as shown in **Table 14**.

**Table 14:** Ablation study for transformer-based SegNet (SegFormer-B0).

Learning Rate	Optimizer	Loss	Epochs	Dice score	IoU
*5e-5*	*Adam*	*CrossEntropy*	*10*	*0.932*	*0.894*
5e-5	AdamW	CrossEntropy	10	0.917	0.878
1e-4	Adam	Dice + BCE	10	0.910	0.871
1e-4	SGD	CrossEntropy	10	0.895	0.859

In **Table 14**, a Dice score of 0.932 and an IoU of 0.894 were obtained using a learning rate of 5e-5, the Adam optimizer, and CrossEntropy loss.

### 4.5 Limitations and future directions

**Figure 12:**
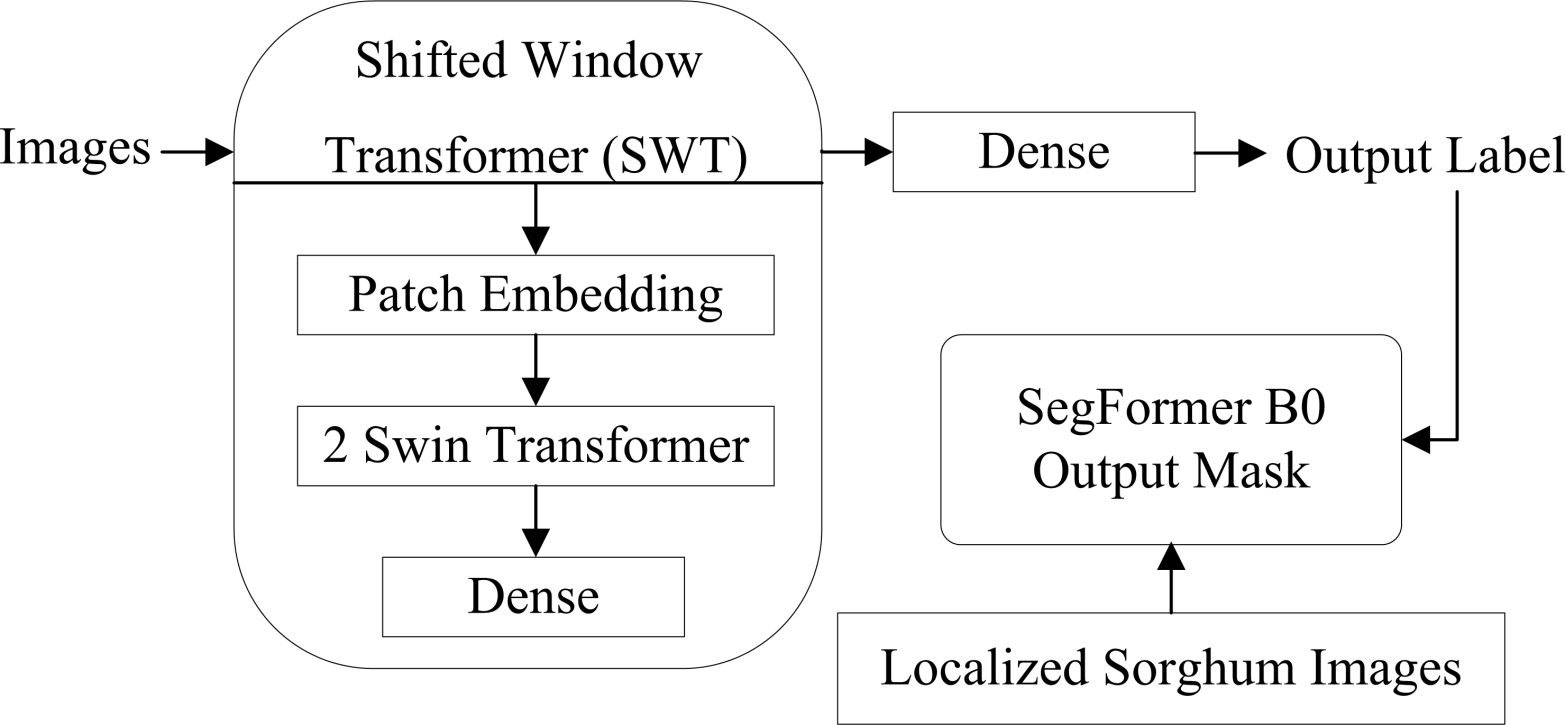
Structure of the proposed method.

The working of the proposed method is given in **Figure 12**.

One limitation of the proposed method is the risk of overfitting, particularly given the training on a relatively small dataset of 4K-resolution sorghum images. High-resolution data can cause the model to memorize fine details rather than generalize well, particularly when the dataset size is not sufficiently large or diverse. Additionally, environmental variations such as lighting, shadow, and noise in the images can further challenge model robustness.

To address this, future validation will involve cross-dataset testing under varying sorghum field conditions and seasons, along with the application of regularization techniques to improve generalizability.

## 5 Conclusion

The process of detecting sorghum crops is challenging due to the variability in shape, size, and texture of the sorghum leaves as well as the limited research that has been conducted so far. During image acquisition, noise and illumination are also significant challenges in sorghum leaves, which degrade the algorithm’s detection accuracy. To overcome the existing challenges, three models are proposed to classify, localize, and segment the sorghum leaves in noisy and illuminated images, which is a big challenge. A shifted window transformer neural network is proposed, based on selected layers and hyperparameters, to classify the different types of sorghum leaves. The results are computed on a publicly available sorghum weed classification dataset with an accuracy of 1.00. The localization of sorghum leaves is still a challenging task; four datasets that were prepared at Kansas State University are publicly available, such as SGH, sorghum leaf detection, sorghum panicle, and sorghum grain head. The YOLOv9-c model is designed on the optimal hyperparameters and trained from scratch for 100 epochs. The model more accurately localized the sorghum leaves and provided a mAP of 0.898, 0.961, and 0.996, respectively. Segmenting sorghum leaves is a challenging task due to the presence of noisy and illuminated images. To address this challenge, a Segformer transformer neural network is proposed and trained from scratch using optimal hyperparameters, which yields an IoU of 0.8973 and a Dice score of 0.9459. The proposed models have shown better performance compared to the existing approaches in this domain. This study constitutes a significant contribution to the field and provides a solid foundation for future scholars to build upon and further develop. This technique may be expanded into a real-time application in the future and made available to the general public for wider application.

Although the proposed model demonstrates high accuracy in classifying sorghum-related images, its transferability to other cereal crops such as wheat, maize, or rice remains unexplored. The current approach is optimized and fine-tuned specifically for sorghum, and without empirical evidence, it is uncertain whether a similar performance can be achieved across different crop types with varying morphological features and disease patterns. This limitation restricts the model’s broader applicability in real-world agricultural scenarios. To overcome this, future work should focus on evaluating the model’s generalization capabilities by testing it on datasets from other cereal crops. Domain adaptation techniques, transfer learning, or multi-crop training strategies could also be incorporated to improve the model’s versatility and ensure a consistent performance across diverse agricultural conditions.

In the future, overfitting and environmental variation will be addressed through semi-supervised learning to leverage unlabeled data and improve generalization. Additionally, domain adaptation techniques will be explored to enhance model robustness across diverse field conditions and unseen environments, strengthening real-world applicability.

## Data Availability

The original contributions presented in the study are included in the article/supplementary material. Further inquiries can be directed to the corresponding author.
